# Bis{μ-2,2′-[1,1′-(ethane-1,2-diyldinitrilo)diethyl­idyne]diphenolato-κ^5^ *O*,*N*,*N*′,*O*′:*O*}bis­[chloridomanganese(III)]

**DOI:** 10.1107/S1600536808000226

**Published:** 2008-01-09

**Authors:** V. S. Thampidas, T. Radhakrishnan, Robert D. Pike

**Affiliations:** a Department of Chemistry, SN College, Varkala, Kerala 695 145, India; b Department of Chemistry, University of Kerala, Thiruvananthapuram, Kerala 695 581, India; c Department of Chemistry, College of William and Mary, PO Box 8795, Williamsburg, VA 23187-8795, USA

## Abstract

The title compound, [Mn_2_(C_18_H_18_N_2_O_2_)_2_Cl_2_], was synthesized by the reaction between manganese(II) *o*-chloro­benzoate and the Schiff base generated *in situ* by the condensation of ethane-1,2-diamine and *o*-hydroxy­acetophenone. The centrosymmetric dimer contains two Jahn–Teller-distorted mangan­ese(III) ions, each in an octa­hedral geometry, connected through two phen­oxy bridges from two ligands.

## Related literature

For related literature, see: Christou (2005 ▶); Horwitz *et al.* (1995 ▶); Jacobsen *et al.* (1991 ▶); Larrow & Jacobsen (2004 ▶); Panja *et al.* (2003 ▶); Pecoraro & Butler (1986 ▶); Saha *et al.* (2004 ▶); Triller *et al.* (2002 ▶); Vites & Lynam (1998 ▶); Zhang *et al.* (1990 ▶).
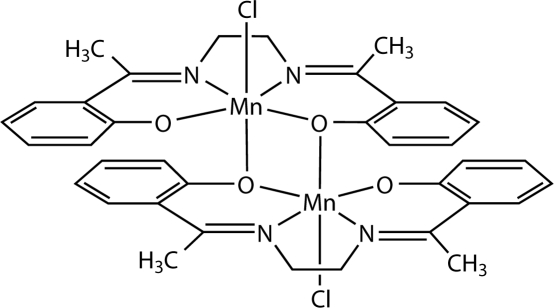


## Experimental

### 

#### Crystal data

[Mn_2_(C_18_H_18_N_2_O_2_)_2_Cl_2_]
*M* *_r_* = 769.47
Triclinic, 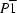

*a* = 7.8261 (3) Å
*b* = 9.8046 (3) Å
*c* = 11.2372 (4) Å
α = 97.207 (2)°
β = 94.701 (2)°
γ = 108.081 (2)°
*V* = 806.49 (5) Å^3^
*Z* = 1
Cu *K*α radiation
μ = 8.29 mm^−1^
*T* = 100 (2) K
0.28 × 0.14 × 0.14 mm

#### Data collection

Bruker SMART APEXII CCD diffractometer
Absorption correction: multi-scan (*SAINT-Plus*; Bruker, 2004 ▶) *T*_min_ = 0.205, *T*_max_ = 0.390 (expected range = 0.165–0.313)
12822 measured reflections
2781 independent reflections
2733 reflections with *I* > 2σ(*I*)
*R*_int_ = 0.033

#### Refinement

*R*[*F* ^2^ > 2σ(*F* ^2^)] = 0.032
*wR*(*F* ^2^) = 0.088
*S* = 1.10
2781 reflections
219 parameters
H-atom parameters constrained
Δρ_max_ = 0.39 e Å^−3^
Δρ_min_ = −0.44 e Å^−3^

### 

Data collection: *APEX2* (Bruker, 2004 ▶); cell refinement: *SAINT-Plus* (Bruker, 2004 ▶); data reduction: *SAINT-Plus*; program(s) used to solve structure: *SHELXS97* (Sheldrick, 2008 ▶); program(s) used to refine structure: *SHELXL97* (Sheldrick, 2008 ▶); molecular graphics: *ORTEP-3* (Farrugia, 1997 ▶); software used to prepare material for publication: *SHELXL97* and *PRPKAPPA* (Ferguson, 1999 ▶).

## Supplementary Material

Crystal structure: contains datablocks global, I. DOI: 10.1107/S1600536808000226/hy2115sup1.cif

Structure factors: contains datablocks I. DOI: 10.1107/S1600536808000226/hy2115Isup2.hkl

Additional supplementary materials:  crystallographic information; 3D view; checkCIF report

## Figures and Tables

**Table:** 

Mn1—O2	1.8738 (16)
Mn1—O1	1.9191 (16)
Mn1—N1	1.9964 (19)
Mn1—N2	2.0129 (19)
Mn1—Cl1	2.4633 (6)
Mn1—O1^i^	2.4720 (16)

**Table:** 

O2—Mn1—O1	95.14 (7)
O2—Mn1—N1	170.58 (8)
O1—Mn1—N1	87.86 (7)
O2—Mn1—N2	90.36 (7)
O1—Mn1—N2	163.69 (8)
N1—Mn1—N2	84.44 (8)
O2—Mn1—Cl1	95.48 (5)
O1—Mn1—Cl1	96.66 (5)
N1—Mn1—Cl1	93.03 (6)
N2—Mn1—Cl1	98.09 (6)
O2—Mn1—O1^i^	88.58 (6)
O1—Mn1—O1^i^	77.02 (7)
N1—Mn1—O1^i^	83.39 (7)
N2—Mn1—O1^i^	87.80 (7)
Cl1—Mn1—O1^i^	172.81 (4)
Mn1—O1—Mn1^i^	102.98 (7)

Symmetry code: (i) 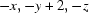.

**Fig. 1.**
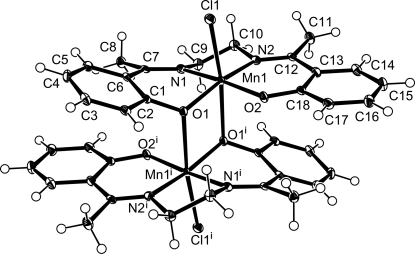
Molecular structure of the title compound. Displacement ellipsoids are drawn at the 50% probability level. [Symmetry code: (i) -x, 2 - y, -z.]

## supplementary crystallographic information

### Comment

Manganese coordination chemistry, in recent decades, has been in intense research focus in connection with developments in diverse fields as bioinorganic modeling (Triller *et al.*, 2002), asymmetric catalysis (Larrow & Jacobsen, 2004), molecular magnetism (Christou, 2005) *etc*. Schiff base ligands with nitrogen and oxygen donor atoms seem to stabilize the various oxidation states of manganese better than any other ligand systems, as it is evident from the sheer number of publications in this area (Vites & Lynam, 1998). The penta-coordinate [Mn(salen)Cl] (H_2_salen = *N*,*N*'-bis(salicylidene)-1,2-diaminoethane) was one of the earliest crystallographically characterized manganese(III) Schiff base complexes (Pecoraro & Butler, 1986). This may be considered as a prototype molecule, that has led to the development of large number of manganese(III) complexes with a square planar MnN_2_O_2_ core, stabilized by a chiral-salen ligand and a chloride ion in the axial position (Zhang *et al.*, 1990; Jacobsen *et al.*, 1991). In our effort to synthesize dimeric manganese(III) complexes of a salen-like ligand, *N*,*N*'-bis(*o*-hydroxyacetophenonylidene)-1,2-diaminoethane with *o*-chlorobenzoate as an ancillary ligand, we unexpectedly obtained a dimeric manganese(III) complex stabilized by the Schiff base and two axial chloride ligands. Here we report the crystal structure of the new dichloride dimer (Fig. 1).

In the title compound, the centrosymmetric dimer is crystallographically half independent and consists of two Mn^III^ atoms, linked by two phenolic O atoms of two ligands. Two Mn—N bonds and two Mn—O bonds complete the equatorial square plane geometry around the Mn^III^ atom (Table 1). This leaves the two axial positions open for coordination to the Cl atoms, leading to the formation of a rare dichloride dimer. Jahn-Teller distortion elongates the Mn—Cl bond [Mn1—Cl1 = 2.4633 (6) Å] substantially, which is comparable to the Mn—Cl bond length of 2.461 (1)Å in the square pyramidal [Mn(salen)Cl] (Pecoraro & Butler, 1986). But the elongation is not as much as seen in the square pyramidal [Mn(5—Cl-salen)Cl] (Horwitz *et al.*, 1995) and octahedral [Mn(salen)Cl(H_2_O)] (Panja *et al.*, 2003), where Mn—Cl distances are 2.572 (1) Å and 2.621 (6) Å, respectively. Jahn-Teller effect is also apparent in the longer Mn—O bond [Mn1—O1 = 2.4720 (16) Å] of the Mn_2_(µ-O)_2_ diamond core of the dimer. This makes the Mn—O—Mn bridge of the complex considerably weaker than that in the diazide dimer [Mn_2_(*L*)_2_(N_3_)_2_] (H_2_*L* = *N*,*N*'-bis(*o*-hydroxyacetophenonylidene)-1,2-diaminoethane) (Saha *et al.*, 2004), where the corresponding bond length is 2.375 (5) Å. The Mn···Mn separation in the title compound is 3.453 (2) Å, compared to 3.341 (2)Å in [Mn_2_(*L*)_2_(N_3_)_2_].

### Experimental

To a solution of Mn(o—Cl—C_6_H_4_CO_2_)_2_.2H_2_O (1.00 g, 2.49 mmol) and *o*-hydroxyacetophenone (0.68 g, 4.98 mmol) in methanol (40 ml), ethane-1,2-diamine (0.14 g, 2.49 mmol) was added. The solution was stirred for 20 min, filtered and left to evaporation in an open conical flask. Brown crystals were deposited in 2–3 days. These were collected by filtration, washed with methanol, and dried in air (yield 0.80 g, 80.6% based on Mn).

### Refinement

H atoms were positioned geometrically and refined as riding atoms, with C—H = 0.95 (CH) and 0.99Å (CH_2_) and *U*_iso_(H) = 1.2*U*_eq_(C), and with C—H = 0.98Å (CH_3_) and *U*_iso_(H) = 1.5*U*_eq_(C).

### Crystal data

**Table:**

[Mn_2_(C_18_H_18_N_2_O_2_)_2_Cl_2_]	*Z* = 1
*M**_r_* = 769.47	*F*_000_ = 396
Triclinic, *P*1	*D*_x_ = 1.584 Mg m^−^^3^
Hall symbol: -P 1	Cu *K*α radiation λ = 1.54178 Å
*a* = 7.8261 (3) Å	Cell parameters from 2781 reflections
*b* = 9.8046 (3) Å	θ = 9.8–71.6º
*c* = 11.2372 (4) Å	µ = 8.29 mm^−^^1^
α = 97.207 (2)º	*T* = 100 (2) K
β = 94.701 (2)º	Prism, brown
γ = 108.081 (2)º	0.28 × 0.14 × 0.14 mm
*V* = 806.49 (5) Å^3^	

### Data collection

**Table:**

Bruker SMART APEXII CCD diffractometer	2781 independent reflections
Radiation source: fine-focus sealed tube	2733 reflections with *I* > 2σ(*I*)
Monochromator: graphite	*R*_int_ = 0.033
*T* = 100(2) K	θ_max_ = 67.0º
φ and ω scans	θ_min_ = 4.0º
Absorption correction: multi-scan(SAINT-Plus; Bruker, 2004)	*h* = −9→9
*T*_min_ = 0.205, *T*_max_ = 0.390	*k* = −11→11
12822 measured reflections	*l* = −13→12

### Refinement

**Table:**

Refinement on *F*^2^	Secondary atom site location: difference Fourier map
Least-squares matrix: full	Hydrogen site location: inferred from neighbouring sites
*R*[*F*^2^ > 2σ(*F*^2^)] = 0.032	H-atom parameters constrained
*wR*(*F*^2^) = 0.088	*w* = 1/[σ^2^(*F*_o_^2^) + (0.0396*P*)^2^ + 1.0982*P*] where *P* = (*F*_o_^2^ + 2*F*_c_^2^)/3
*S* = 1.10	(Δ/σ)_max_ = 0.001
2781 reflections	Δρ_max_ = 0.39 e Å^−^^3^
219 parameters	Δρ_min_ = −0.44 e Å^−^^3^
Primary atom site location: structure-invariant direct methods	Extinction correction: none

### Fractional atomic coordinates and isotropic or equivalent isotropic displacement parameters (Å^2^)

**Table:**

	*x*	*y*	*z*	*U*_iso_*/*U*_eq_	
Mn1	0.03321 (5)	0.93493 (4)	0.13283 (3)	0.00849 (13)	
Cl1	0.23277 (7)	0.82169 (6)	0.23406 (5)	0.01447 (15)	
O1	0.1568 (2)	0.96398 (17)	−0.00716 (14)	0.0108 (3)	
O2	0.1517 (2)	1.12309 (17)	0.21677 (14)	0.0118 (3)	
N1	−0.1287 (3)	0.7452 (2)	0.04014 (18)	0.0107 (4)	
N2	−0.1560 (3)	0.9047 (2)	0.24675 (17)	0.0109 (4)	
C18	0.1226 (3)	1.1893 (3)	0.3192 (2)	0.0114 (5)	
C12	−0.1614 (3)	0.9876 (3)	0.3457 (2)	0.0107 (5)	
C5	0.1437 (3)	0.6051 (3)	−0.1622 (2)	0.0137 (5)	
H5	0.0687	0.5063	−0.1770	0.016*	
C7	−0.0921 (3)	0.6659 (2)	−0.0498 (2)	0.0112 (5)	
C8	−0.2364 (3)	0.5291 (3)	−0.1141 (2)	0.0142 (5)	
H8A	−0.2528	0.4528	−0.0634	0.021*	
H8B	−0.1993	0.4970	−0.1910	0.021*	
H8C	−0.3509	0.5488	−0.1297	0.021*	
C2	0.3629 (3)	0.8946 (3)	−0.1229 (2)	0.0119 (5)	
H2	0.4379	0.9934	−0.1109	0.014*	
C1	0.2018 (3)	0.8569 (2)	−0.0705 (2)	0.0102 (4)	
C11	−0.3108 (3)	0.9334 (3)	0.4225 (2)	0.0160 (5)	
H11A	−0.4250	0.9375	0.3830	0.024*	
H11B	−0.2802	0.9946	0.5022	0.024*	
H11C	−0.3238	0.8328	0.4321	0.024*	
C9	−0.3112 (3)	0.7098 (3)	0.0770 (2)	0.0135 (5)	
H9A	−0.3738	0.6036	0.0577	0.016*	
H9B	−0.3829	0.7588	0.0323	0.016*	
C13	−0.0262 (3)	1.1327 (3)	0.3837 (2)	0.0123 (5)	
C14	−0.0442 (3)	1.2227 (3)	0.4882 (2)	0.0158 (5)	
H14	−0.1439	1.1874	0.5315	0.019*	
C6	0.0872 (3)	0.7094 (3)	−0.0912 (2)	0.0106 (4)	
C17	0.2500 (3)	1.3270 (3)	0.3664 (2)	0.0141 (5)	
H17	0.3542	1.3622	0.3270	0.017*	
C10	−0.2963 (3)	0.7598 (3)	0.2119 (2)	0.0139 (5)	
H10A	−0.4146	0.7652	0.2331	0.017*	
H10B	−0.2637	0.6889	0.2569	0.017*	
C3	0.4149 (3)	0.7907 (3)	−0.1920 (2)	0.0138 (5)	
H3	0.5247	0.8183	−0.2269	0.017*	
C16	0.2281 (3)	1.4120 (3)	0.4680 (2)	0.0165 (5)	
H16	0.3142	1.5054	0.4963	0.020*	
C4	0.3054 (3)	0.6445 (3)	−0.2101 (2)	0.0146 (5)	
H4	0.3426	0.5726	−0.2555	0.018*	
C15	0.0780 (4)	1.3594 (3)	0.5286 (2)	0.0184 (5)	
H15	0.0604	1.4177	0.5976	0.022*	

### Atomic displacement parameters (Å^2^)

**Table:**

	*U*^11^	*U*^22^	*U*^33^	*U*^12^	*U*^13^	*U*^23^
Mn1	0.0089 (2)	0.0085 (2)	0.0068 (2)	0.00102 (14)	0.00293 (13)	0.00013 (14)
Cl1	0.0154 (3)	0.0175 (3)	0.0120 (3)	0.0072 (2)	0.0016 (2)	0.0030 (2)
O1	0.0127 (8)	0.0106 (8)	0.0091 (8)	0.0036 (6)	0.0037 (6)	0.0006 (6)
O2	0.0130 (8)	0.0121 (8)	0.0087 (8)	0.0018 (6)	0.0039 (6)	0.0004 (6)
N1	0.0095 (9)	0.0113 (9)	0.0110 (10)	0.0017 (7)	0.0032 (7)	0.0036 (8)
N2	0.0104 (9)	0.0115 (9)	0.0110 (10)	0.0028 (8)	0.0027 (7)	0.0031 (8)
C18	0.0136 (11)	0.0143 (11)	0.0085 (11)	0.0074 (9)	0.0005 (9)	0.0026 (9)
C12	0.0108 (11)	0.0160 (11)	0.0078 (11)	0.0072 (9)	0.0015 (8)	0.0042 (9)
C5	0.0182 (12)	0.0103 (11)	0.0109 (11)	0.0027 (9)	0.0004 (9)	0.0008 (9)
C7	0.0159 (12)	0.0108 (11)	0.0068 (11)	0.0040 (9)	−0.0004 (9)	0.0029 (9)
C8	0.0142 (12)	0.0132 (11)	0.0129 (12)	0.0017 (9)	0.0029 (9)	0.0004 (9)
C2	0.0119 (11)	0.0135 (11)	0.0089 (11)	0.0027 (9)	−0.0011 (9)	0.0021 (9)
C1	0.0121 (11)	0.0128 (11)	0.0061 (10)	0.0052 (9)	−0.0004 (8)	0.0011 (9)
C11	0.0154 (12)	0.0188 (12)	0.0129 (12)	0.0037 (10)	0.0059 (9)	0.0014 (10)
C9	0.0094 (11)	0.0138 (11)	0.0154 (12)	0.0012 (9)	0.0037 (9)	0.0011 (9)
C13	0.0139 (12)	0.0150 (11)	0.0087 (11)	0.0056 (9)	0.0014 (9)	0.0015 (9)
C14	0.0166 (12)	0.0207 (12)	0.0108 (12)	0.0064 (10)	0.0044 (9)	0.0023 (10)
C6	0.0117 (11)	0.0136 (11)	0.0062 (10)	0.0041 (9)	−0.0009 (8)	0.0015 (9)
C17	0.0143 (12)	0.0150 (11)	0.0121 (11)	0.0029 (9)	0.0028 (9)	0.0029 (9)
C10	0.0119 (11)	0.0134 (11)	0.0148 (12)	0.0005 (9)	0.0057 (9)	0.0039 (9)
C3	0.0117 (11)	0.0199 (12)	0.0096 (11)	0.0049 (9)	0.0027 (9)	0.0009 (9)
C16	0.0194 (13)	0.0137 (11)	0.0135 (12)	0.0028 (10)	0.0005 (9)	−0.0010 (10)
C4	0.0169 (12)	0.0154 (12)	0.0123 (12)	0.0078 (10)	0.0021 (9)	−0.0021 (9)
C15	0.0242 (13)	0.0196 (13)	0.0117 (12)	0.0089 (11)	0.0035 (10)	−0.0021 (10)

### Geometric parameters (Å, °)

**Table:**

Mn1—O2	1.8738 (16)	C8—H8C	0.9800
Mn1—O1	1.9191 (16)	C2—C3	1.385 (3)
Mn1—N1	1.9964 (19)	C2—C1	1.400 (3)
Mn1—N2	2.0129 (19)	C2—H2	0.9500
Mn1—Cl1	2.4633 (6)	C1—C6	1.423 (3)
Mn1—O1^i^	2.4720 (16)	C11—H11A	0.9800
O1—C1	1.348 (3)	C11—H11B	0.9800
O1—Mn1^i^	2.4720 (16)	C11—H11C	0.9800
O2—C18	1.321 (3)	C9—C10	1.517 (3)
N1—C7	1.302 (3)	C9—H9A	0.9900
N1—C9	1.469 (3)	C9—H9B	0.9900
N2—C12	1.305 (3)	C13—C14	1.422 (3)
N2—C10	1.483 (3)	C14—C15	1.378 (4)
C18—C17	1.413 (3)	C14—H14	0.9500
C18—C13	1.424 (3)	C17—C16	1.382 (3)
C12—C13	1.471 (3)	C17—H17	0.9500
C12—C11	1.513 (3)	C10—H10A	0.9900
C5—C4	1.379 (4)	C10—H10B	0.9900
C5—C6	1.418 (3)	C3—C4	1.402 (3)
C5—H5	0.9500	C3—H3	0.9500
C7—C6	1.467 (3)	C16—C15	1.398 (4)
C7—C8	1.510 (3)	C16—H16	0.9500
C8—H8A	0.9800	C4—H4	0.9500
C8—H8B	0.9800	C15—H15	0.9500
			
O2—Mn1—O1	95.14 (7)	O1—C1—C2	118.2 (2)
O2—Mn1—N1	170.58 (8)	O1—C1—C6	122.4 (2)
O1—Mn1—N1	87.86 (7)	C2—C1—C6	119.3 (2)
O2—Mn1—N2	90.36 (7)	C12—C11—H11A	109.5
O1—Mn1—N2	163.69 (8)	C12—C11—H11B	109.5
N1—Mn1—N2	84.44 (8)	H11A—C11—H11B	109.5
O2—Mn1—Cl1	95.48 (5)	C12—C11—H11C	109.5
O1—Mn1—Cl1	96.66 (5)	H11A—C11—H11C	109.5
N1—Mn1—Cl1	93.03 (6)	H11B—C11—H11C	109.5
N2—Mn1—Cl1	98.09 (6)	N1—C9—C10	109.25 (19)
O2—Mn1—O1^i^	88.58 (6)	N1—C9—H9A	109.8
O1—Mn1—O1^i^	77.02 (7)	C10—C9—H9A	109.8
N1—Mn1—O1^i^	83.39 (7)	N1—C9—H9B	109.8
N2—Mn1—O1^i^	87.80 (7)	C10—C9—H9B	109.8
Cl1—Mn1—O1^i^	172.81 (4)	H9A—C9—H9B	108.3
C1—O1—Mn1	121.87 (14)	C14—C13—C18	117.7 (2)
C1—O1—Mn1^i^	112.89 (13)	C14—C13—C12	119.4 (2)
Mn1—O1—Mn1^i^	102.98 (7)	C18—C13—C12	122.9 (2)
C18—O2—Mn1	130.34 (15)	C15—C14—C13	122.2 (2)
C7—N1—C9	121.6 (2)	C15—C14—H14	118.9
C7—N1—Mn1	127.36 (16)	C13—C14—H14	118.9
C9—N1—Mn1	110.67 (14)	C5—C6—C1	118.3 (2)
C12—N2—C10	119.38 (19)	C5—C6—C7	119.8 (2)
C12—N2—Mn1	129.33 (16)	C1—C6—C7	121.7 (2)
C10—N2—Mn1	111.08 (14)	C16—C17—C18	122.1 (2)
O2—C18—C17	116.5 (2)	C16—C17—H17	118.9
O2—C18—C13	125.0 (2)	C18—C17—H17	118.9
C17—C18—C13	118.5 (2)	N2—C10—C9	109.88 (18)
N2—C12—C13	121.5 (2)	N2—C10—H10A	109.7
N2—C12—C11	119.1 (2)	C9—C10—H10A	109.7
C13—C12—C11	119.4 (2)	N2—C10—H10B	109.7
C4—C5—C6	121.3 (2)	C9—C10—H10B	109.7
C4—C5—H5	119.4	H10A—C10—H10B	108.2
C6—C5—H5	119.4	C2—C3—C4	119.9 (2)
N1—C7—C6	120.7 (2)	C2—C3—H3	120.1
N1—C7—C8	119.9 (2)	C4—C3—H3	120.1
C6—C7—C8	119.4 (2)	C17—C16—C15	119.4 (2)
C7—C8—H8A	109.5	C17—C16—H16	120.3
C7—C8—H8B	109.5	C15—C16—H16	120.3
H8A—C8—H8B	109.5	C5—C4—C3	119.9 (2)
C7—C8—H8C	109.5	C5—C4—H4	120.0
H8A—C8—H8C	109.5	C3—C4—H4	120.0
H8B—C8—H8C	109.5	C14—C15—C16	119.8 (2)
C3—C2—C1	121.3 (2)	C14—C15—H15	120.1
C3—C2—H2	119.4	C16—C15—H15	120.1
C1—C2—H2	119.4		
			
O2—Mn1—O1—C1	144.83 (16)	Mn1—N1—C7—C8	−176.24 (16)
N1—Mn1—O1—C1	−44.12 (17)	Mn1—O1—C1—C2	−145.77 (17)
N2—Mn1—O1—C1	−105.9 (3)	Mn1^i^—O1—C1—C2	90.9 (2)
Cl1—Mn1—O1—C1	48.69 (16)	Mn1—O1—C1—C6	37.8 (3)
O1^i^—Mn1—O1—C1	−127.83 (18)	Mn1^i^—O1—C1—C6	−85.5 (2)
O2—Mn1—O1—Mn1^i^	−87.35 (7)	C3—C2—C1—O1	−178.2 (2)
N1—Mn1—O1—Mn1^i^	83.71 (7)	C3—C2—C1—C6	−1.7 (3)
N2—Mn1—O1—Mn1^i^	21.9 (3)	C7—N1—C9—C10	149.6 (2)
Cl1—Mn1—O1—Mn1^i^	176.51 (4)	Mn1—N1—C9—C10	−36.7 (2)
O1^i^—Mn1—O1—Mn1^i^	0.0	O2—C18—C13—C14	−175.6 (2)
O1—Mn1—O2—C18	172.36 (19)	C17—C18—C13—C14	4.1 (3)
N2—Mn1—O2—C18	7.7 (2)	O2—C18—C13—C12	3.3 (4)
Cl1—Mn1—O2—C18	−90.43 (19)	C17—C18—C13—C12	−177.0 (2)
O1^i^—Mn1—O2—C18	95.52 (19)	N2—C12—C13—C14	176.1 (2)
O1—Mn1—N1—C7	25.1 (2)	C11—C12—C13—C14	−3.6 (3)
N2—Mn1—N1—C7	−169.3 (2)	N2—C12—C13—C18	−2.9 (3)
Cl1—Mn1—N1—C7	−71.4 (2)	C11—C12—C13—C18	177.4 (2)
O1^i^—Mn1—N1—C7	102.3 (2)	C18—C13—C14—C15	−1.2 (4)
O1—Mn1—N1—C9	−148.16 (15)	C12—C13—C14—C15	179.8 (2)
N2—Mn1—N1—C9	17.44 (15)	C4—C5—C6—C1	0.1 (3)
Cl1—Mn1—N1—C9	115.28 (14)	C4—C5—C6—C7	175.1 (2)
O1^i^—Mn1—N1—C9	−70.98 (15)	O1—C1—C6—C5	178.0 (2)
O2—Mn1—N2—C12	−7.3 (2)	C2—C1—C6—C5	1.6 (3)
O1—Mn1—N2—C12	−117.2 (3)	O1—C1—C6—C7	3.1 (3)
N1—Mn1—N2—C12	−179.4 (2)	C2—C1—C6—C7	−173.3 (2)
Cl1—Mn1—N2—C12	88.3 (2)	N1—C7—C6—C5	161.2 (2)
O1^i^—Mn1—N2—C12	−95.9 (2)	C8—C7—C6—C5	−20.0 (3)
O2—Mn1—N2—C10	178.04 (15)	N1—C7—C6—C1	−24.0 (3)
O1—Mn1—N2—C10	68.1 (3)	C8—C7—C6—C1	154.8 (2)
N1—Mn1—N2—C10	5.90 (15)	O2—C18—C17—C16	175.2 (2)
Cl1—Mn1—N2—C10	−86.38 (15)	C13—C18—C17—C16	−4.5 (4)
O1^i^—Mn1—N2—C10	89.48 (15)	C12—N2—C10—C9	157.5 (2)
Mn1—O2—C18—C17	172.84 (15)	Mn1—N2—C10—C9	−27.3 (2)
Mn1—O2—C18—C13	−7.4 (3)	N1—C9—C10—N2	41.7 (3)
C10—N2—C12—C13	−179.6 (2)	C1—C2—C3—C4	−0.1 (4)
Mn1—N2—C12—C13	6.2 (3)	C18—C17—C16—C15	1.8 (4)
C10—N2—C12—C11	0.2 (3)	C6—C5—C4—C3	−1.9 (4)
Mn1—N2—C12—C11	−174.11 (16)	C2—C3—C4—C5	1.9 (4)
C9—N1—C7—C6	175.2 (2)	C13—C14—C15—C16	−1.5 (4)
Mn1—N1—C7—C6	2.5 (3)	C17—C16—C15—C14	1.2 (4)
C9—N1—C7—C8	−3.6 (3)		

Symmetry codes: (i) −*x*, −*y*+2, −*z*.
